# Microbial Musings – December 2020

**DOI:** 10.1099/mic.0.001019

**Published:** 2020-12-23

**Authors:** Gavin H. Thomas

**Affiliations:** ^1^​ Department of Biology, University of York, York, YO10 5YW, UK

As we end this extraordinary year, the importance of microbiology continues to be demonstrated in our daily lives in ways that we would never have predicted 12 months ago. This month in the UK, the identification of a new variant of severe acute respiratory syndrome coronavirus 2 (SARS-CoV-2) through the continuing work of the COVID-19 Genomics Consortium (@CovidGenomicsUK) led to an almost immediate change in government policy, and while the manifestations of the new variant are being uncovered, it is an example of evolution occurring in real time in front of the eyes of the general public. The development and delivery of multiple new vaccines against the virus are a true illustration that biology has the potential to be the greatest technology of the 21st century.

As we look forward to the situation improving slowly across the globe through 2021, I want to highlight some exciting changes at the journal as we move into its 74th year. One problem with having a journal with a title that is literally as broad as the whole subject, is that sometime authors can feel that they should look for a more specialized publication. To address this, we have entirely reworked the topic areas in which we are looking for submissions. From January 2021 we will have 10 new topic areas (Table 1), which are aimed to help identify and build new communities around the journal and the Microbiology Society. To support this move, we have appointed four new Senior Editors to help our leadership team to lead and develop these communities. For our ‘Microbial cell surfaces’ topic we have Tracy Palmer FRS from the University of Newcastle, and for our ‘Microbial evolution’ topic we have Mike Brockhurst from the University of Liverpool, while Steve Diggle from Georgia Tech will lead the ‘Microbial interactions and communities’ topic and Martin Welch, University of Cambridge, will co-lead the large ‘Microbial physiology, biochemistry and metabolism’ topic. I am delighted to bring these new faces into our senior team to bring the journal to its 75th anniversary and beyond. Also, three of the topic areas relate to areas of research where the Microbiology Society has identified clear roles for our subject in delivering solutions to global problems by linking them to the United Nations Sustainable Development Goals (UN SDGs). We have mapped the ‘circular economy’ to our ‘Biotechnology and synthetic biology’ topic, ‘soil health’ to our ‘Plant microbiology and soil health’ topic and ‘antimicrobial resistance (AMR)’ to our ‘Antimicrobials and AMR’ theme. Next month I will be announcing a significant number of new editors to handle submissions within our revised topic areas.

**Table 1.** New topic areas for *Microbiology*

New topic area
Antimicrobials and AMR (UN SDG 'AMR')
Biotechnology and synthetic biology (UN SDG 'circular economy')
Cell and developmental microbiology
Microbial cell surfaces
Microbial evolution
Microbial interactions and communities
Microbial physiology, biochemistry and metabolism
Microbial virulence and pathogenesis
Plant microbiology and soil health (UN SDG 'Soil health')
Regulation, sensing and signalling

The first paper from this issue to highlight is one that would fit nicely into the new Plant microbiology and soil health topic, as it is around the biology of two *Bacillus* species strains that are currently used as biocontrol agents in China, and more specifically are used to protect oil seed rape crop from a pathogenic fungal infections. The work from Alex Mullins (@AlexJMullins), collaborating with Eshwar Mahenthiralingam and Gordon Webster at the University of Cardiff, UK, and colleagues at the Oil Crops Research Institute of Chinese Academy of Agricultural Sciences, Wuhan, PR China, aimed to use genome sequencing to learn more about these strains, and the authors were immediately able to place them into the species *Bacillus velezensis* [1]. With this information to hand, Mullins and colleagues compared the genomes of their strains with those of a large number of other *B. velezensis* strains and noted the precociousness of their strains for producing natural products. Experimentally they demonstrated that the lipopeptide iturin A, which both their strains have the biosynthetic cluster for, can kill the fungi that their strains protect against, making this a likely component of the biocontrol process. However, they suspect that their strains produce a mixture of natural products that they could see encoded on their genomes, some of which have been detected previously biochemically [2], which may indicate why this species more generally produces such good biocontrol agents.

The next two papers concern bacteria of the genus *Vibrio *, both the classical pathogenic species *V. cholerae* and the gastrointestinal pathogen *V. vulnificus* [3, 4]. In the first paper from Nabendu Chatterjee’s group at the National Institute of Cholera and Enteric Diseases, Kolkata, India [3], the authors further characterize the *V. cholerae* transcription factor CytR, which is already known from the work of Brian Hammer’s (@briankhammer) group at Georgia Tech, USA, to regulate competence, some type VI secretion systems and chitinase gene expression [5]. In this work the authors find that a *cytR* mutant has an additional defect in growth on porcine mucin as the sole source of carbon, which is also correlated with observed reductions in motility and mucin penetration. Some of the known chitinases are thought to be secreted enzymes involved in the breakdown of small *N*-acetylglucosamine (GlcNAc) containing oligosaccharides released from mucin [6], so this could also explain part of this phenotype. They also find the mutant is less able to adhere to intestinal epithelia and has a colonization defect in a suckling mouse model. These additional functions, alongside its established functions, demonstrate that CytR is an important regulator of *V. cholerae* pathogenesis. The second *Vibrio* paper focuses on the foodborne pathogen *V. vulnificus *, a nasty bug with a high fatality rate in the USA. The study by Laura Cutugno (@LauraCutugno) from the group of Aoife Boyd, with Conor O’Byrne (@cpobyrne) at the University of Galway, Ireland, looked at the responses of the well-studied clinical strain CMCP6 when this is selected for rifampicin resistance [4]. This is known to occur clinically, although fortunately these bacteria are still sensitive to most antibiotics, and from isolating independent genetic lines with this phenotype, which results in mutations in *rpoB*, they were able to show a wide range of pleiotropic phenotypes, which varied considerably with the nature of the allele of *rpoB*, with only reduced motility being a common phenotype. While this is known from similar genotype/phenotype studies in other bacteria, it is relevant in *V. vulnificus *, as rifampicin resistance is often used as a genetic marker and could be having quite different effects on cellular physiology that are being overlooked.

There are two methods papers in this issue, a format that I hope will be used more in the future, and which I would like to highlight here in the *Musings*. The first is a paper from the group of Daniel Daley at Stockholm University, Sweden. Daley’s surname is one that is etched in my mind from a paper he published as a postdoc with Gunnar von Heijne, whose group at the time were applying computational and experimental methods to learn the fundamental rules of membrane protein biosynthesis, folding and function. I had spent a long time a few years earlier when a postdoc at the John Innes Centre (JIC) in Norwich, UK, determining the experimental topology of the ammonium transporter AmtB, using fusions to location-specific reporters [7]. After all this painstaking work, the experimental mapping was pretty much what I had predicted from bioinformatics applying principles such as the positive inside rule described by Gunnar in 1986 [8], which then agreed with the structure published a few years later [9]. Daniel then comes along and builds on the strength of these computational models to use a single C-terminal GFP tag fused separately to all of the 601 *Escherichia coli* inner membrane proteins and determine a complete inner membrane protein topology map! [10]. This kind of blew my mind and I remember spending a lot of time reading and annotating these data for *Echo*BASE [11] as the first genome-wide experimental study of protein structure. Anyway, I digress. The paper in this issue from Bill Söderström (@BillSoderstrom) and Alessandro Ruda, with colleagues from the Daley group, describes a new fluorescently labelled d-alanine for labelling peptidoglycan, which has improved functionality for use in the most recent iterations of microscopy that can be applied to small bacterial cells [12]. The new label uses OreganGreen488 as the fluorophore, which has higher resistance to photobleaching and is more photo-stable than other commonly used fluorophores. This is important for new super-resolution methods such as 3D structured illumination microscopy (3D-SIM) and stimulated emission depletion (STED), which are now being applied to bacteria cells [13]. They demonstrate that their new label is efficiently incorporated into peptidoglycan, first in a range of Gram-positive bacteria, and compare this to a similar fluorescein-based label, where the new label is able to resolve at much higher resolution. Using a pulse of their new label followed by another of a different colour, they observe labelling of new peptidoglycan at the division site and then this moving away as the cell grows. As the new molecule, OGDA, is ~500 Da, it cannot label *E. coli* very efficiently as it needs to get into the cell to be built into the peptidoglycan precursor molecule, although they did see good labelling in the biotechnologically relevant Gram-negative bacterium *Zymomonas mobilis *. This is an exciting new development and no doubt Söderström will be using this in his new laboratory at the ithree institute (@ithreeinst) in Sydney, Australia.

The second methods paper is from Emma Holden from the group of Eleftheria Trampari, with colleagues Gregory Wickham (@gwickh) and Mark A Webber (@ma_webber) from the Quadram Institute, Norwich, UK [14]. This presents a simple, easy-to-use two-plasmid system for introducing genes quickly onto the genomes of *Enterobacteriaceae *, exemplified in this work by *Salmonella enterica* serovar Typhimurium, where the authors exploit the widely used lambda Red recombinase system [15] for the insertion of DNA at a neutral Tn*7* site downstream of the *glmS* gene, a site has been used for neutral insertions in several other *Enterobacteriaceae *. The system uses the pDOC-K plasmids [16] developed for recombineering in strains that cannot be easily transformed with linear double-stranded DNA, which are modified here to enable homologous recombination with the Tn*7* recognition site. The authors test the system by knocking in a series of genes at this locus and they show here that it has no fitness cost in *S*. Typhimurium. The vectors have been deposited in Addgene for all to use.

The final paper to highlight with a strong methods component is one by Esther Sweeney (@SweeneyEsther) from the group of Freya Harrison (@friendlymicrobe) at the University of Warwick, UK, in collaboration with Andrew Edwards (@bugsinblood) at Imperial College, London. In this work the authors use a fluorescently labelled antibiotic to look at its penetration, or rather lack of penetration, into *Pseudomonas aeruginosa* [17], and you can read about this in the Microbiology Society blog, as it has been selected as Editors’s Choice by Senior Editor Jen Cavet.

We close with an interesting review on Nudix proteins and their role in bacterial pathogenesis from Elzbieta Kraszewska and Joanna Drabinska at the Polish Academy of Science in Warsaw, Poland [18]. These proteins, exemplified by *E. coli* MutT, were first characterized as nucleoside triphosphatases that stop the cell incorporating oxidized bases into their DNA and RNA. Their substrate range is, however, much greater than just 8-oxo-GTP, and these hydrolases can also act on mRNA targets functioning as 5′-triphosphatases to produce a 5′-monophosphate that can be attacked by ribonucleases, thus influencing mRNA stability [19]. As well as fundamental functions within bacterial cells, they also come into play as genuine pathogenicity factors, as some Nudix proteins are secreted into host cells, where they disrupt host processes, such as the RipN effector from *Ralstonia solanacearum* that alters the NADH/NAD^+^ ratio in plant cells [20], and they are also used by the oomycete pathogen *Phytophthora* to modify the plant immune system [21]. Interestingly, Nudix domain variants exist where the active site has been mutated and their basic nucleoside-binding function is used as a domain within a family of transcription factors, which are called Nudix-related transcriptional regulators (NrtRs) [22], the function of which have been confirmed experimentally [23].

Enjoy a short respite over the festive period and let’s get ready for a challenging, but hopefully ultimately positive 2021.

## References

[1] Mullins AJ, Li Y, Qin L, Hu X, Xie L (2020). Reclassification of the biocontrol agents *Bacillus subtilis* BY-2 and Tu-100 as *Bacillus velezensis* and insights into the genomic and specialized metabolite diversity of the species. Microbiology.

[2] Rabbee MF, Ali MS, Choi J, Hwang BS, Jeong SC (2019). *Bacillus velezensis:* A Valuable Member of Bioactive Molecules within Plant Microbiomes. Molecules.

[3] Das S, Chourashi R, Mukherjee P, Gope A, Koley H (2020). Multifunctional transcription factor CytR of *Vibrio cholerae* is important for pathogenesis. Microbiology.

[4] Cutugno L, Mc Cafferty J, Pané-Farré J, O'Byrne C, Boyd A (2020). *rpoB* mutations conferring rifampicin-resistance affect growth, stress response and motility in *Vibrio vulnificus*. Microbiology.

[5] Watve SS, Thomas J, Hammer BK (2015). Cytr is a global positive regulator of competence, type VI secretion, and chitinases in Vibrio cholerae. PLoS One.

[6] Mondal M, Nag D, Koley H, Saha DR, Chatterjee NS (2014). The Vibrio cholerae extracellular chitinase ChiA2 is important for survival and pathogenesis in the host intestine. PLoS One.

[7] Thomas GH, Mullins JG, Merrick M (2000). Membrane topology of the Mep/Amt family of ammonium transporters. Mol Microbiol.

[8] Heijne G (1986). The distribution of positively charged residues in bacterial inner membrane proteins correlates with the trans-membrane topology. Embo J.

[9] Khademi S, O'Connell J, Remis J, Robles-Colmenares Y, Miercke LJW (2004). Mechanism of ammonia transport by Amt/MEP/Rh: structure of AmtB at 1.35 a. Science.

[10] Daley DO, Rapp M, Granseth E, Melén K, Drew D (2005). Global topology analysis of the Escherichia coli inner membrane proteome. Science.

[11] Misra RV, Horler RSP, Reindl W, Goryanin II, Thomas GH (2005). EchoBASE: an integrated post-genomic database for Escherichia coli. Nucleic Acids Res.

[12] Söderström B, Ruda A, Widmalm G, Daley DO (2020). An OregonGreen488-labelled D-amino acid for visualizing peptidoglycan by super-resolution STED nanoscopy. Microbiology.

[13] Söderström B, Chan H, Daley DO (2019). Super-Resolution images of peptidoglycan remodelling enzymes at the division site of Escherichia coli. Curr Genet.

[14] Holden ER, Wickham GJ, Webber MA, Thomson NM, Trampari E (2020). Donor plasmids for phenotypically neutral chromosomal gene insertions in *Enterobacteriaceae*. Microbiology.

[15] Datsenko KA, Wanner BL (2000). One-Step inactivation of chromosomal genes in Escherichia coli K-12 using PCR products. Proc Natl Acad Sci U S A.

[16] Lee DJ, Bingle LEH, Heurlier K, Pallen MJ, Penn CW (2009). Gene doctoring: a method for recombineering in laboratory and pathogenic Escherichia coli strains. BMC Microbiol.

[17] Sweeney E, Sabnis A, Edwards AM, Harrison F (2020). Effect of host-mimicking medium and biofilm growth on the ability of colistin to kill *Pseudomonas aeruginosa*. Microbiology.

[18] Kraszewska E, Drabinska J (2020). Nudix proteins affecting microbial pathogenesis. Microbiology.

[19] Deana A, Celesnik H, Belasco JG (2008). The bacterial enzyme RppH triggers messenger RNA degradation by 5' pyrophosphate removal. Nature.

[20] Sun Y, Li P, Shen D, Wei Q, He J (2019). The Ralstonia solanacearum effector RipN suppresses plant PAMP-triggered immunity, localizes to the endoplasmic reticulum and nucleus, and alters the NADH/NAD^+^ ratio in Arabidopsis. Mol Plant Pathol.

[21] Dong S, Yin W, Kong G, Yang X, Qutob D (2011). Phytophthora sojae avirulence effector Avr3b is a secreted NADH and ADP-ribose pyrophosphorylase that modulates plant immunity. PLoS Pathog.

[22] Rodionov DA, De Ingeniis J, Mancini C, Cimadamore F, Zhang H (2008). Transcriptional regulation of NAD metabolism in bacteria: NrtR family of Nudix-related regulators. Nucleic Acids Res.

[23] Gao R, Wei W, Hassan BH, Li J, Deng J (2019). A single regulator NrtR controls bacterial NAD^+^ homeostasis via its acetylation. Elife.

